# Estimated size of the clinical medical imaging physics workforce in the United States

**DOI:** 10.1002/acm2.13664

**Published:** 2022-06-14

**Authors:** Sean D. Rose, David W. Jordan, Nicholas B. Bevins, Jaydev K. Dave, David E. Hintenlang, Brad K. Lofton, Pankaj Patel

**Affiliations:** ^1^ University of Texas Health Science Center at Houston Houston Texas USA; ^2^ University Hospitals Cleveland Medical Center Cleveland Ohio USA; ^3^ Case Western Reserve University Cleveland Ohio 44106 USA; ^4^ Henry Ford Health System Detroit Michigan USA; ^5^ Thomas Jefferson University Philadelphia Pennsylvania USA; ^6^ The Ohio State University Columbus Ohio USA; ^7^ Colorado Associates in Medical Physics Colorado Springs Colorado USA

**Keywords:** modeling, supply, workforce

## Abstract

There is no current authoritative accounting of the number of clinical imaging physicists practicing in the United States. Information about the workforce is needed to inform future efforts to secure training pathways and opportunities. In this study, the AAPM Diagnostic Demand and Supply Projection Working Group collected lists of medical physicists from several state registration and licensure programs and the Conference of Radiation Control Program Directors (CRCPD) registry. By cross‐referencing individuals among these lists, we were able to estimate the current imaging physics workforce in the United States by extrapolating based on population. The imaging physics workforce in the United States in 2019 consisted of approximately 1794 physicists supporting diagnostic X‐ray (1073 board‐certified) and 934 physicists supporting nuclear medicine (460 board‐certified), with a number of individuals practicing in both subfields. There were an estimated 235 physicists supporting nuclear medicine exclusively (150 board‐certified). The estimated total workforce, accounting for overlap, was 2029 medical physicists. These estimates are in approximate agreement with other published studies of segments of the workforce.

## 1 INTRODUCTION

The size of the imaging physics workforce (encompassing clinical medical physics support for diagnostic and interventional imaging and nuclear medicine)^1^ in the United States is not definitively known and is challenging to determine accurately. Details about the present workforce are needed as initial conditions for future projections of medical physicist supply and workforce needs (demand). There is a public health interest in developing and maintaining an adequate workforce of medical physicists.^2^ Some data and reviews suggest potential future shortages of trained medical physicists^3^; meanwhile, there has been recent debate about the roles and viability of the various training and experience pathways governing entry to the profession. Of note, imaging physicists comprise a smaller portion of the medical physicist workforce than radiation therapy physicists and are affected by different professional and economic dynamics.

Over the past decade, there has been substantial financial investment by the American Association of Physicists in Medicine (AAPM), Radiological Society of North America (RSNA), and Society of Nuclear Medicine and Molecular Imaging (SNMMI) in grants to create new imaging physics residency programs and training positions. These grants have supported a proliferation of residencies from the small number that existed around 2014 when the American Board of Radiology (ABR) first required candidates for initial certification in medical physics to complete residency training programs accredited by the Commission on Accreditation of Medical Physics Education Programs (CAMPEP). As of 2020, there are 29 CAMPEP‐accredited imaging physics residency programs, and it is not clear whether the current output of these programs will be "right‐sized" to the job market or will result in an oversupply or shortage of imaging physicists. As both the size of the medical physics workforce and the number of residency programs have grown, there is now an interest in supply and demand modeling to better quantify the true size of the needed workforce.^4^

The AAPM established the Diagnostic Demand and Supply Projection Working Group in 2019 to gather data from a variety of sources, develop and validate demand and supply models, and prepare future projections of the training and employment landscape for medical imaging physicists. In this study, the Working Group members collected and synthesized publicly available data to estimate the size of the clinical imaging physics workforce as of 2019 and compared current and historical data with projections made in the past and results of other recent estimates.

## 2 METHODS

### 2.1 Data collection approach

There is no single comprehensive, authoritative source available as a catalog or census of the current medical imaging physics workforce in the United States. There are a number of available data sources; each has limitations as an accurate representation of the workforce. We combined several data sources in an attempt to create a reasonable estimate of the size of the current United States clinical imaging physicist workforce.

As of 2019, AAPM membership was about 8700 individuals working in medical physics or closely related fields. Database details can be used to exclude those who reside (and thus likely work) outside the United States, as well as to separate retirees and trainees based on their membership category. One limitation is that members' work activities are self‐reported, so it is not feasible to determine consistently their clinical medical physics subspecialties (e.g., diagnostic, therapy, or both). Further, clinical physicists cannot be reliably distinguished from those working in research, regulatory, or other nonclinical functions (which, while crucial segments of the imaging physics workforce, do not require clinical residency training). Retirement is also a confounder as some retirees choose to maintain full membership rather than transitioning to Emeritus membership. Yet another limitation is that an unknown number of individuals who are not AAPM members work in clinical imaging physics.

The annual AAPM Professional Information survey (also known as the salary survey) garners a high response rate (greater than 40% of AAPM members). This survey collects current information about respondents' employment, practice subspecialty or subspecialties, and mix of clinical and nonclinical work. Respondents are self‐selected and the survey is not designed for representative sampling, so it is likely that extrapolation of the survey results to nonrespondents and to non‐AAPM members in the workforce would be inaccurate.

The Conference of Radiation Control Program Directors (CRCPD) Qualified Medical Physicist (QMP) Registry is updated frequently (at least annually) with lists of medical physicists currently certified by the ABR, American Board of Medical Physics (ABMP), American Board of Science in Nuclear Medicine (ABSNM), and Canadian College of Physicists in Medicine (CCPM), and health physicists certified by the American Board of Health Physics (ABHP). Each listing contains the home or work address provided by the individual to the respective certifying board. The certification category (medical physics subspecialty) is provided in the listing. Individuals holding multiple certifications have multiple listings (one for each certificate). There are two limitations to using this list for workforce estimates. First, the list of currently certified physicists may contain entries for individuals who are retired and no longer actively working. Second, the list is likely incomplete because there are various pathways to practice medical imaging physics that do not require board certification. However, this list provides a complete and up‐to‐date list of individuals currently certified by the included medical physics specialty boards.

Individual states’ radiation control programs have their own required qualifications to practice medical physics, which range from licensure to state certification or accreditation to registration to no requirements. Some states have optional programs, such that some practicing individuals are registered with the state while others choose not to be. Many states require approval for X‐ray imaging physics services but not for nuclear medicine. State credentials typically require renewal every 1–3 years, so state lists are not likely to contain very many retired individuals, assuming that only those actively working would continue to renew their state credentials. State listings usually include both board‐certified and noncertified individuals, so they are a potential source of information to estimate the number of noncertified medical physicists in the workforce. On a nationwide basis, a union of individual states’ lists would contain many duplicates, as imaging physicists commonly work in multiple states and obtain credentials in each.

Although many imaging physicists who hold board certification, state credentials, or both support MRI and ultrasound in addition to ionizing radiation modalities, there are some whose clinical practice is exclusively in MRI or ultrasound and who do not hold state credentials. The latter group is not included in this workforce supply estimate as we were unable to locate any reliable data sources to count or identify them.

### 2.2 State workforce estimates

To estimate the imaging physics workforce size, the working group obtained a current copy of the CRCPD registry data (as of April 2020) and state imaging physicist licensee or registrant lists from California, Colorado, Florida, Illinois, Indiana, Maryland, Massachusetts, New Jersey, New York, Ohio, and Texas. Table 1 provides a summary of the data collected from each state.

**TABLE 1:** Description of qualifications of individuals listed in state imaging physicist directories and criteria for counting individuals from each list in national diagnostic X‐ray and nuclear medicine physicist workforce estimates. Temporary licensees in Texas were included because under Texas regulations they can work under general supervision of a full licensee

State	Data	Criteria for inclusion in nuclear medicine physicist supply estimate	Criteria for inclusion in diagnostic (X‐ray) physicist supply estimate
CA	Individuals authorized to conduct mammography surveys [17 CCR § 30315.52]	N/A	All included
CO	Individuals approved to perform evaluations of radiation machines, facilities, and operators for compliance. [6 CCR 1007‐1 Part 02, 2.4.4.1]	N/A	Qualified inspectors authorized to perform certification evaluations in CT, fluoroscopy, or mammography.
FL	Individuals with temporary or full licensure in either Diagnostic Radiological Physics or Medical Nuclear Physics. This includes all individuals able to provide nuclear or radiological physics services [FS Title XXXII § 483.901]	Full licensure in medical nuclear physics.	Full licensure in diagnostic radiological physics.
IL	Registered diagnostic imaging specialists in mammography and/or general radiography. Hospitals performing CT or mammography are required to have a radiation protection program overseen by such an individual [32 Ill. Adm. Code 410]	N/A	All included
IN	List of approved physicists and inspectors. Routine testing of diagnostic X‐ray equipment must be performed by these individuals [410 IAC 5–6.1‐118(c)]	N/A	Individuals qualified as diagnostic imaging physicists or X‐ray machine inspectors.
MD	List of state licensed private inspectors. This license allows individuals to inspect X‐ray equipment as part of the state's certification process. Inspectors can additionally be approved to perform physics services in mammography, but the data did not indicate which inspectors had this additional approval [COMAR 26.12.02].	N/A	All included
MA	List of individuals registered to perform health physics services in the areas of (1) diagnostic radiology (excluding mammography) and (2) mammography. These individuals are able to perform health physics consultations or surveys in these areas. [105 CMR 120]	N/A	All included
NJ	Qualified medical physicists for the supervision of quality assurance programs for computed tomography, diagnostic X‐ray, and/or mammography equipment. [NJAC 7:28‐22.12 and 7:28‐15.4]. Qualified medical physicist assistants in radiography and fluoroscopy were not included.	N/A	All included
NY	Individuals with full licensure in either diagnostic radiological physics or medical nuclear physics. This includes all individuals able to provide nuclear or radiological physics services [8 EDN § 166]	Full licensure in medical nuclear physics	Full licensure in diagnostic radiological physics
OH	Opt‐in lists of certified radiation experts available to perform cone beam CT testing and shielding design and area surveys. Certified radiation experts can serve as the individual responsible for radiation protection for an imaging provider [Ohio Adm. Code 3701:1‐66‐03]	N/A	All included
TX	Individuals with temporary or full licensure in either Diagnostic Radiological Physics or Medical Nuclear Physics. This includes all individuals able to provide nuclear or radiological physics services [22 TAC §160]	Temporary or full licensure in Medical Nuclear Physics	Temporary or full licensure in Diagnostic Radiological Physics

Because of variations in regulatory requirements, registration or licensure data from each state contain different subpopulations of the imaging physics workforce. For example, Texas licensure data contain all physicists authorized to provide nuclear medicine or diagnostic imaging physics services, whereas California's registration data only contain individuals authorized to provide mammography physics services. The working group attempted to estimate separately the size of the X‐ray imaging and nuclear medicine physicist supply in each state. Table 1 shows how individuals from the lists were included in the workforce estimates for each state. Although all 11 states provided lists identifying X‐ray imaging physicists, only the data from Florida, New York, and Texas identified nuclear medicine physicists. Therefore, all nuclear medicine workforce estimates in this study are based on the data from these three states.

To create state‐specific workforce estimates that could be extrapolated to a national estimate, we first discarded names from each state list if the listed address was out‐of‐state (to avoid duplication). We then calculated workforce estimates using the criteria in Table 1. To create board‐certified X‐ray imaging or nuclear medicine physics workforce estimates, we first filtered the CRCPD list to individuals with board certifications relevant to X‐ray imaging or nuclear medicine physics, respectively. We then compared the remaining list to each state list using a custom Python program for fuzzy string matching (fuzzywuzzy package). We recorded the number of individuals matching between the state and CRCPD lists as the number of board‐certified physicists in the state. We counted individuals on the state list who did not match to the CRCPD list as nonboard‐certified physicists in the state. Individuals on the CRCPD list with no match on the state list were presumed no longer to be practicing or to have moved out‐of‐state without having updated their certifying board. We took the total workforce for each state to be the sum of the board‐certified and noncertified physicists from this analysis.

### 2.3 Population data

We obtained United States population data for 2019 from the United States Census Bureau.^5^ For each state and for the entire nation, we report the numbers of total and board‐certified imaging physicists as the number of physicists per million people.

### 2.4 National estimates

We used the ratio estimator^6^, ^7^ to calculate national estimates for the total number of X‐ray imaging and nuclear medicine physicists. The estimator is given by

(1) $$n_{US}=n_{sample}\times \frac{x_{US}}{x_{sample}},$$

where $n_{sample}$ is the number of physicists in the sampled states, $x_{US}$ is the population of the United States, and $x_{sample}$ is the population of the sampled states. We chose this estimator because we found the number of imaging physicists per capita to be stable across states.

We calculated the national population‐based estimates for X‐ray imaging physicists using the state estimates for Colorado, Florida, Illinois, Indiana, Maryland, New Jersey, New York, and Texas. We did not include the population‐based estimates for California, Massachusetts, and Ohio in the national estimate because the state listings for these states appear to consist of different basis groups of physicists than those from the included states.

### 2.5 Statistical methods

To quantify the uncertainty in our national workforce estimates, we report an estimate of the standard deviation of the ratio estimator in simple random sampling.^7^ This estimate derives from an experiment in which a fixed number of states are selected at random for data collection, with each state having equal probability of inclusion. The national estimate from such an experiment is a random variable due to the random nature of the sampling. In our study, states were selected based on population, geography, license or registration requirements, and public availability of data. This is most appropriately modeled as a deterministic process, so strictly speaking, a standard deviation does not exist. We adopted the standard deviation estimate based on simple random sampling as a means to convey the approximate uncertainty in our workforce size estimates.

### 2.6 Other models

In 2009, the Center for Health Workforce Studies (CHWS) at SUNY Albany published a study of the medical physics workforce on behalf of AAPM, including modeling and future projections.^8^ A key aim of this work was to understand the likely impact of changes to ABR certification eligibility in 2012 and 2014, and, in particular, the potential consequences of the new requirement for residency training beginning in 2014. CHWS conducted a survey of AAPM members and, based on the results, estimated that in 2009, there were 769 clinical medical imaging physicists in the United States. They assumed equilibrium between supply and demand at that time and projected that future demand would scale proportionally to the size of the U.S. population. Future supply projections (based on varying rates of opening new residency slots) were compared to these demand curves to estimate the number of additional residency training slots that would be needed by the early 2020s to meet the projected demand for imaging physicists.

AAPM and SNMMI published a joint task force report in 2015^9^ assessing the state of nuclear medicine physics training. This report discussed current and future estimates of the U.S. nuclear medicine workforce as context for the adequate availability of clinical training opportunities.

We used estimates from both reports as comparisons in this work.

## 3 RESULTS

### 3.1 Population‐Based estimates of X‐ray imaging physicists

The results of the list reconciliation for individual states, together with state population figures and per capita workforce estimates for X‐ray physicists, are listed in Table 2.

**TABLE 2:** X‐ray imaging physicists for each state based on state and CRCPD listings

State	# Physicists on CRCPD registry	# Physicists on state list	# Board‐certified physicists in state	2019 state population	Total physicists per million residents	Board‐certified physicists per million residents
CO	36	36	27	5 758 736	6.25	4.69
FL	46	88	69	21 477 737	4.10	3.21
IL	51	86	35	12 671 821	6.79	2.76
IN	16	42	12	6 732 219	6.24	1.78
MD	36	59	22	6 751 429	8.74	3.26
NJ	52	52	37	8 882 190	5.85	4.17
NY	103	104	69	19 453 561	5.35	3.55
TX	129	138	91	28 995 881	4.76	3.14
CA	123	73	49	39512 223	1.85	1.24
MA	34	41	23	6 892 503	5.95	3.34
OH	75	42	19	11 689 100	3.59	1.63

As of March 2020, the CRCPD registry contained 1322 unique names associated with one or more certifications relevant to diagnostic imaging physics. The national estimate of Board‐certified X‐ray imaging physicists (based on cross‐referencing state and CRCPD lists and extrapolating to the U.S. population) is 1073 (*SD *= 52). The total estimated X‐ray imaging medical physics workforce is 1794 individuals (*SD *= 134) which includes both board‐certified and nonboard‐certified physicists.

**FIGURE 1:**
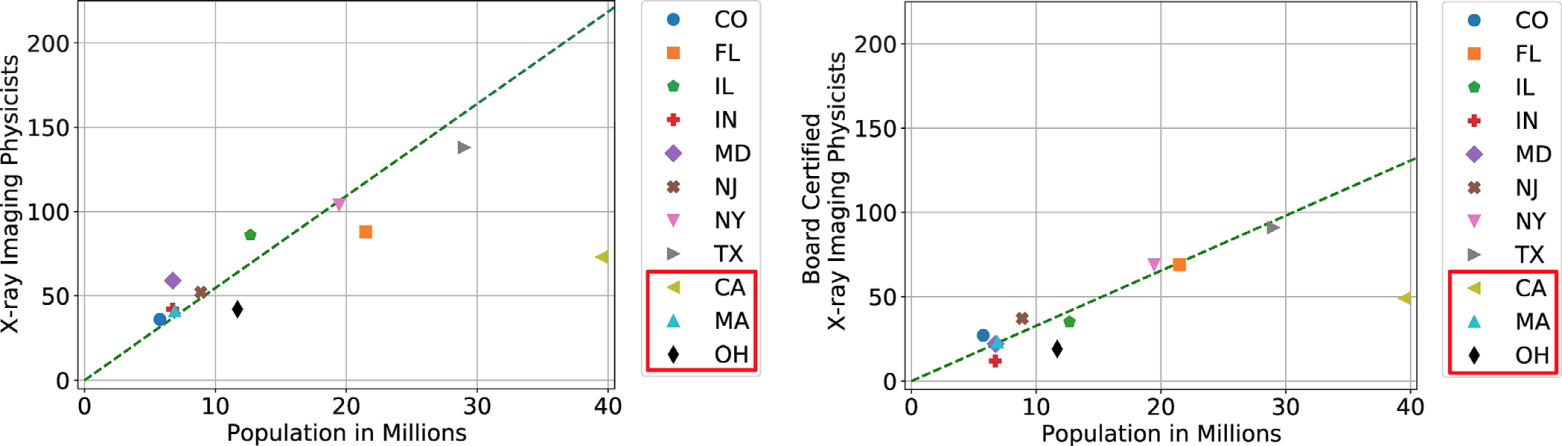
Number of total and board‐certified X‐ray imaging physicists in each state versus state population. Data points for California, Massachusetts, and Ohio are plotted but not included in the population‐based model. The dashed green line has slope equal to the per capita population‐based workforce size estimate. This line, if extended to the total U.S. population size, would indicate our estimate of the total national workforce

Based on the 2019 U.S. Census data, the population‐based supply estimate is 5.5 X‐ray imaging physicists per million U.S. residents, with about 60% of these board‐certified. Figure 1 shows the distribution of individual states’ X‐ray imaging physicists and populations and the average per‐capita population‐based workforce size estimate.

Using the CRCPD registry alone would overestimate the supply of board‐certified medical physicists active in clinical service, which agrees with observations of members of the working group who are personally familiar with retired physicists whose names still appear on the CRCPD registry.

### 3.2 Population‐based estimates of nuclear medicine physicists

The results of the list reconciliation for individual states, together with state population figures and per capita workforce estimates for nuclear medicine physicists, are listed in Table 3.

**TABLE 3:** Nuclear medicine physicists for each state based on state and CRCPD listings

Who is counted	State	# Physicists on CRCPD Registry	# Physicists on state list	# Board‐certified physicists in state	2019 state population	Total physicists per million residents	Board‐certified physicists per million residents
Nuclear medicine exclusive physicists	FL		19	10	21 477 737	0.88	0.47
	NY		25	20	19 453 561	1.29	1.03
	TX		6	2	28 995 881	0.21	0.07
All nuclear medicine physicists	FL	19	51	27	21 477 737	2.37	1.26
	NY	67	68	36	19 453 561	3.50	1.85
	TX	70	80	35	28 995 881	2.76	1.21

**FIGURE 2:**
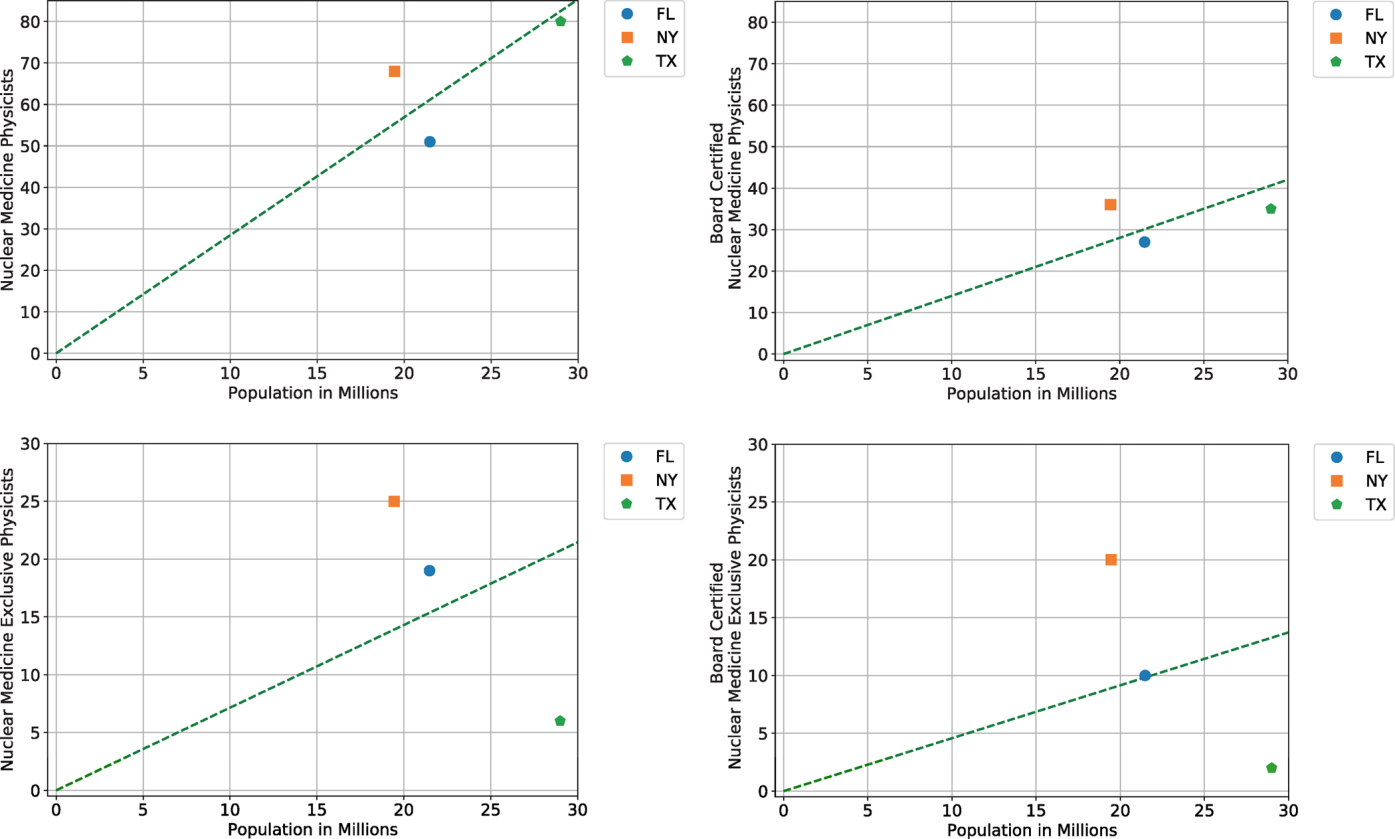
Number of total and board‐certified nuclear medicine physicists in each state versus state population. Panels (a) and (b) show the total number of physicists practicing nuclear medicine, whereas Panels (c) and (d) show the number of physicists credentialed in nuclear medicine but not in other medical physics disciplines. The dashed green line has slope equal to the per capita population‐based workforce size estimate

As of 2019, the CRCPD registry contained 636 individuals with certifications relevant to nuclear medicine physics. Among the states we reviewed, only those with licensure requirements (FL, NY, and TX) require state approval to practice nuclear medicine physics. After matching the CRCPD list to these state lists and performing a similar population analysis and extrapolation, the national estimate of board‐certified nuclear medicine physicists is 460 (*SD *= 60), and the national estimate of the total number of nuclear medicine physicists is 934 (*SD *= 91). The total supply is currently estimated at 2.8 nuclear medicine physicists per million people, with 49% of these board‐certified. The top two panels of Figure 2 show the distribution of individual states’ nuclear medicine physicists and populations and the average per capita population‐based workforce size estimate.

As there are many nuclear medicine physicists who also perform X‐ray physics services, we additionally calculated a national estimate for the number of physicists providing nuclear medicine services exclusively. The national estimate of board‐certified nuclear medicine exclusive physicists is 150 (*SD *= 88), and the national estimate of the total number of nuclear‐medicine‐exclusive physicists is 235 (*SD *= 105). The total supply is currently estimated at 0.7 exclusively‐nuclear‐medicine physicists per million people, with 64% of these board‐certified. The bottom two panels of Figure 2 show the distribution of individual states’ nuclear medicine exclusive physicists and populations and the average per‐capita population‐based workforce size estimate.

### 3.3 Estimated size and composition of the imaging physics workforce

**FIGURE 3:**
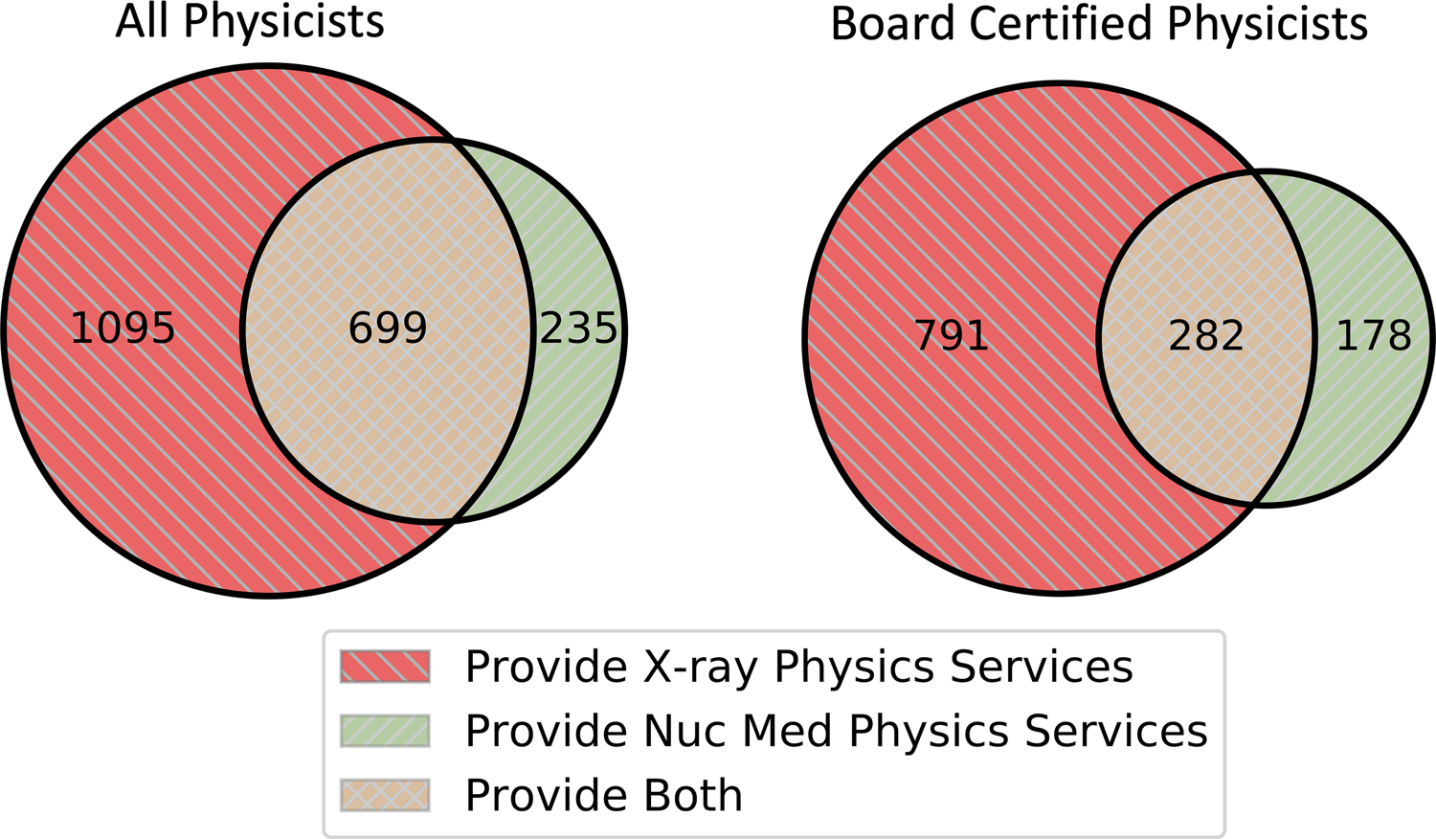
Venn diagram illustrating the subgroups of imaging physicists who provide clinical services supporting diagnostic X‐ray, nuclear medicine, or both. This summarizes the size and composition of the workforce because we collected diagnostic X‐ray and nuclear medicine physicist credentials separately while many individuals within the workforce are active in both areas. The board‐certified diagram represents physicists who are board certified in at least one of the subspecialties in which they practice (e.g., some physicists in the green region support both X‐ray and nuclear medicine, but they are board certified in only nuclear medicine). This is why the count in the green region differs from the estimate of board‐certified physicists exclusively supporting nuclear medicine reported in the text

A substantial number of imaging physicists support both diagnostic X‐ray and nuclear medicine. To illustrate the overlap between these groups, we provide a Venn diagram (Figure 3) of the subgroups that we estimate to be actively supporting one or both subfields. The total workforce consists of an estimated 2029 medical physicists. We estimate 1251 of these physicists are board certified in at least one of the subfields they actively support.

## 4 DISCUSSION

The CHWS study estimated a baseline supply of 769 diagnostic medical physicists in 2009 for a U.S. population of 307 212 000 people,^10^ or 2.5 imaging physicists per million people; they projected that this number would remain flat or decline slightly through 2020, depending on the number of imaging physics residency positions that would become available between 2014 and 2022. Considering a simple population‐based supply projection rather than residency‐constrained scenarios, a constant supply ratio of 2.5 imaging physicists per million people would have predicted a total workforce of 821 diagnostic physicists in 2019. A limitation of the CHWS study was that it estimated the workforce size from the responses to an AAPM member survey. This method has known limitations acknowledged in the study report, and the report does not provide the estimated uncertainties for these figures. It is possible that the 2009 figures underestimated the true workforce size at that time.

The CHWS projected demand values for 2019 were 807–849 total imaging physicists, allowing for uncertainty in the actual rate of demand and demand growth from 2009 to 2019, reflecting a shortage of imaging physicists by 2019 if supply were constrained by the number of available residency training slots.^8^

The joint AAPM‐SNMMI task force estimated the national workforce to consist of 340 board‐certified nuclear medicine physicists as of 2015.^9^ That study did not estimate the size of the noncertified nuclear medicine physicist workforce.

The individual state population‐based estimates for California, Massachusetts, and Ohio were not used in calculating the national population‐based estimate because they contain different basis groups of medical physicists than the data collected from the other states in this study. The mammography listing for California contains a much smaller number of individuals relative to the state's population than other states’ lists, reflecting the fact that many other individuals who provide services for radiography, fluoroscopy, and CT (among other types of equipment) are not included in the list. The Massachusetts list is a list of registered consultants; it was not clear from our review whether it reflects a complete list of individuals authorized to practice medical physics or an opt‐in listing of those who wish to offer their services to the public. The Massachusetts list also contains both the names of individuals and of companies. It was not clear to us whether the individual physicist employees of these companies would appear individually on the list. The Ohio list is not a complete list of qualified diagnostic physicists either; it is a subset consisting of individuals who register with the state as Certified Radiation Experts (optional for qualified individuals) and who also opt‐in to be included on the state‐provided referral lists. The state provides these lists to X‐ray facility registrants who need medical physicist services to correct common inspection noncompliance findings. Thus, the workforce estimated from the Ohio list was not appropriate for extrapolation to other states based on population.

For the remaining states, the lists obtained represent the physicists authorized to perform medical physics evaluations of all types of X‐ray equipment. We considered these representative, such that extrapolations from these states could be used to estimate the workforces in other states based on each state's population and to make a population‐based estimate of the national workforce size. We obtained registration lists from states with a range of geographic sizes and population densities to create a population‐based estimate that would be valid for the national workforce across all states.

Prior to this study, the CHWS study^8^ was the most recent comprehensive assessment of the imaging physics workforce in the United States. The CHWS supply projections are substantially smaller than the supply we estimated for 2019. The CHWS supply projection model assumed, however, that the pathway for nonboard‐certified physicists to enter the profession would close completely by around 2020. This model disregarded the large number of nonboard‐certified individuals who readily qualify to serve the major demand drivers for imaging physics services and the fact that in 2020, it is still possible to enter the field, and remain in it permanently, via these pathways. Future investigations of the imaging physics service market and demand conditions will need to include detailed assessment of the nonboard‐certified population and the role of nonboard‐certified pathways into clinical imaging physics practice.

The imaging physics demand projections for 2019 from CHWS are also about a factor of 2 smaller than the estimated workforce size in this study. The future demand projections in the CHWS study used a simple ratio of physicists to total U.S. population and assumed a typical rate of future U.S. population growth. The CHWS projections did not model any changes in the per capita demand rate. However, between 2009 and 2019, there have been several events and trends that likely increased the per capita demand for clinical imaging physics services. In 2012, the Medicare Improvements for Patients and Providers Act (MIPPA) drove a large increase in the demand for accreditation (including imaging physics support) of advanced diagnostic imaging services in nonhospital settings. This was followed by new accreditation requirements implemented by The Joint Commission in 2015 that made medical physics support for advanced diagnostic imaging mandatory in most hospitals and hospital‐operated outpatient facilities. These events help to explain the observation that the size of the medical physics workforce may have increased from around 2.5 diagnostic physicists per million Americans in 2009 to around 6 per million by 2019.

CHWS projected that from 2009 through 2019 and into the early 2020′s, adding 30–50 new imaging physics residency graduates per year to the workforce would still constrain the supply to grow more slowly than demand (in a scenario of relatively modest demand growth). In the intervening years, with the establishment of 29 CAMPEP‐accredited imaging residency programs, it appears that there are likely to be at least 30 new residency graduates per year. However, as the 2019 imaging physics workforce is about twice the size of both the supply and demand projected by CHWS for 2019, additional research is needed to understand the required number of residency programs and slots to support the workforce and serve the needs of patients.

Of note, the workforce growth vastly outpaced CHWS projections over the same period that the growth in residency programs reached about the level that CHWS projected would be required. This and the fact that over one‐third of the current workforce is nonboard‐certified suggest that a substantial number of imaging physicists are entering the workforce via alternate pathways rather than residency and board certification.

There are a number of potential limitations to the accuracy of our estimates. Due to differences among state credentialing programs and accreditation program requirements, we made several assumptions when combining data sources to create individual state estimates and when extrapolating these estimates to national estimates. While we believe that these assumptions are reasonable, they are not necessarily accurate. The total imaging physics workforce comprises a mix of board‐certified and nonboard‐certified individuals. The ratio is different among states due to differing licensure or registration requirements. As our estimates were based on states with defined licensure or registration requirements, the ratio of board‐certified to noncertified physicists we found may not be applicable to states with much more or less stringent requirements. In addition, we focused on counting individual physicists who reside and hold state‐issued medical physics credentials in the same state. While we excluded individuals residing outside our focus states from our counts, these physicists would still be accounted for in the national estimates. We also assumed a kind of equilibrium among physicists working across state lines, on the premise that each state's population requires a fixed amount of physics support. Our analysis assumes that, for example, for each physicist residing out‐of‐state and working in Texas, there would be a Texas‐resident physicist doing a corresponding amount of work outside Texas. While this assumption is likely not valid for many individual examples, for the aggregate, national workforce, it is reasonable to infer the size of the workforce based on the size of the population for whom it provides services.

Medical physicists who work part time rather than full time are included in our data collection and analysis, so a limitation of these estimates is that they would overestimate the full‐time equivalent workforce if there were many semiretired or part‐time working physicists in the states we examined. Many medical physicists who retire gradually reduce their workload over a number of years rather than immediately transitioning from full‐time employment to full retirement. Others work less than full time in clinical service for other reasons, including personal and family leave, job‐sharing arrangements, and substantial effort toward teaching or research efforts. These individuals must maintain their certifications and state credentials while working clinically part time. We expect that the workforce survey conducted by AAPM in parallel with this work will better elucidate the clinical and nonclinical effort levels, near‐term retirement plans, work–life balance considerations of the current imaging physics workforce.

An important area that we do not fully address in this analysis is that of clinical MRI physicists (also called MRI scientists) and ultrasound physicists. Although those physicists who are also qualified to support X‐ray or nuclear medicine provide much MRI and ultrasound physics support, there are some individuals who work in these modalities exclusively, and outside of the four licensure states, there are no state credentials required. Thus, we expect that there exist some number of specialist MRI and ultrasound physicists who are not included in these estimates. As no reliable data sources for these groups could be identified, further research would likely be needed to establish the number of such individuals in practice currently.

## 5 CONCLUSION

The imaging physics workforce in the United States in 2019 consisted of approximately 1794 physicists supporting diagnostic X‐ray (1073 board‐certified) and 934 physicists supporting nuclear medicine (460 board‐certified), with a number of individuals practicing in both subfields. There were an estimated 235 physicists supporting nuclear medicine exclusively (150 board‐certified). The estimated total workforce, accounting for overlap, was 2029 medical physicists. The per capita number of imaging physicists may have increased between 2009 and 2019, likely because of increased demand for medical physics services in support of advanced diagnostic imaging accreditation. The supply of imaging physicists has not been meaningfully constrained by limited availability of residency positions between 2009 and 2019, likely due to continued recognition of nonboard‐certified pathways for new imaging physicists to enter the workforce in support of regulatory and accreditation requirements.

## AUTHOR CONTRIBUTION

DWJ conceived the study design. SDR performed coding, data cleaning, and analysis. All authors contributed to analysis and interpretation of the results. DWJ and SDR drafted the manuscript. All authors contributed to revision of the manuscript and gave final approval.

## DISCLOSURE STATEMENT

The Chair of the Diagnostic Demand and Supply Projection Working Group has reviewed the required Conflict of Interest statement on file for each member and determined that disclosure of potential Conflicts of Interest is an adequate management plan.

The members of the Diagnostic Demand and Supply Projection Working Group listed below attest that they have no potential Conflicts of Interest related to the subject matter or materials presented in this document:

Sean D. Rose

David W. Jordan

Nicholas B. Bevins

Jaydev K. Dave

David E. Hintenlang

Brad K. Lofton

Pankaj Patel

Expenses associated with this work were budgeted and paid by the American Association of Physicists in Medicine on behalf of the Diagnostic Demand and Supply Projection Working Group.

## References

[1] Gress DA , Jordan DW , Butler PF , et al. AAPM Report No. 301. An Updated Description of the Professional Practice of Diagnostic and Imaging Medical Physics: The Report of AAPM Diagnostic Work and Workforce Study Subcommittee. American Association of Physicists in Medicine; May 2017 2017. 301.

[2] CC 2: Meeting the Needs of the Nation for Radiation Protection . National Council on Radiation Protection and Measurements. https://ncrponline.org/program‐areas/cc‐2/ Published 2017. Accessed May 26, 2020.

[3] Newhauser WD . The medical physics workforce. Health Phys. 2017;112(2):139‐148. 28027152 10.1097/HP.0000000000000614

[4] Dempsey C . Medical physics workforce modelling: do we need what we want? Australas Phys Eng Sci Med. 2018;41(3):565‐566. 29961913 10.1007/s13246-018-0663-6

[5] State Population Totals: 2010–2019 . United States Census Bureau. https://www.census.gov/content/census/en/data/datasets/time‐series/demo/popest/2010s‐state‐total.html. Accessed April 29, 2020.

[6] Fuller WA . Sampling Statistics. 1st ed. Wiley; 2009.

[7] Thompson SK . Sampling. 3rd ed. John Wiley & Sons; 2012.

[8] Forte G . Workforce Study of Medical Physicists in the U.S. Center for Health Workforce Studies, SUNY Albany; 2010.

[9] Harkness BA , Allison JD , Clements JB , et al. AAPM/SNMMI Joint Task Force: report on the current state of nuclear medicine physics training. J Appl Clin Med Phys. 2015;16(5):3‐13. 10.1120/jacmp.v16i5.5661 PMC5690174 26699325

[10] Statistical Abstract of the United States: 2009 . United States Census Bureau. https://www.census.gov/library/publications/2008/compendia/statab/128ed.html. Accessed November 23. 2020.

